# The Curious Incident of Crick in the Night-Time and Other Asilomar Enigmas

**DOI:** 10.1007/s10739-025-09805-y

**Published:** 2025-02-26

**Authors:** Matthew Cobb

**Affiliations:** University of Manchester, Manchester, UK

**Keywords:** Asilomar, Paul Berg, Francis Crick, Niels Jerne, Matthew Meselson, Genetic Engineering

## Abstract

In 1971, Paul Berg asked Francis Crick about his views on a controversial proposed experiment involving recombinant DNA; to Berg’s surprise, Crick had no comment to make. This article first describes the multiple reasons why Crick did not respond to Berg, including psychological factors that affected Crick at the time, the limits of his unstated reflexively positivist approach to social issues, and his reluctance to pursue social or political issues when challenged. Crick’s lack of involvement in discussions about recombinant DNA, including in the Asilomar process, is then used to explore two other notable absences from Asilomar: the immunologist Niels Jerne, who was invited to be on the organizing committee but was not involved for reasons that are unclear; and molecular biologist and biological weapons campaigner Matthew Meselson, whose presence would have added a layer of understanding to the discussions, particularly regarding the threat of bioweapons. These enigmatic absences raise questions about the representativeness of Asilomar and suggest future investigations as to how the legacy of Asilomar was shaped both by those who were present–and by those who were not.

“Is there any other point to which you would wish to draw my attention?”

– “To the curious incident of the dog in the night-time.”

“The dog did nothing in the night-time.”

– “That was the curious incident,” remarked Sherlock Holmes.

*The Adventure of Silver Blaze* – Arthur Conan Doyle, 1892.

In June 1971, Bob Pollack of Cold Spring Harbor Laboratory telephoned Paul Berg at Stanford, to ask him why he was planning to transfer DNA from the SV40 virus into *E. coli* (Friedberg 2014). Pollack was alarmed because SV40 could cause uncontrolled growth in hamster cells and there was concern it might be carcinogenic in humans (Pollack and Cobb 2022). After a tense conversation, Berg dismissed Pollack’s worries, but he subsequently discussed the matter with friends and colleagues and recognized that there might be an ethical dimension to his proposed experiment, which he abandoned a few months later.^1^ He continued with the experiment that really interested him, which involved introducing parts of the well-understood lac operon of *E. coli* into SV40; this could then be used to transfect that DNA into a mammalian cell line, where gene function in a eukaryote could be studied for the first time (Jackson et al. 1972). This work contributed to the awarding of Berg’s 1980 Nobel Prize.

The first anyone outside of Berg’s circle knew of his plan and the objections to it was in the summer of 1971, when Berg attended a two-week NATO-sponsored Summer School on Molecular and Developmental Biology in Erice in Sicily (Grunberg-Manago 1989).^2^ Before the school began, the hundred-odd students were asked which topics they wanted to discuss in the informal sessions to be held each evening. Twenty-six students chose to discuss genetic engineering, and a debate duly took place after Berg had presented his proposed experiments. According to John Lear in his 1978 book *Recombinant DNA: The Untold Story*, it went like this:

The young Europeans in the audience reacted volubly. With the ghost of Adolf Hitler still goose-stepping amongst them, they saw themselves on the brink of a frightening new era of human engineering and behaviour control. […] About eighty people came, drank beer, and argued until midnight. “Should we do this or shouldn’t we do it?” Ethical and moral considerations had the floor without interruption. That approach did not appeal to Berg. He would have preferred to discuss the problem in terms of health hazards. But he couldn’t get a word in edgewise. (Lear 1978, p. 37)

But there was something that specifically bemused Berg, according to Lear:

The behaviour of the most eminent person present on the castle top – Sir Francis Crick – fascinated Berg. Crick didn’t say a word all evening. When Berg personally asked the knighted Nobelist for an opinion, he could elicit none. It seemed exceedingly odd to Berg that a man who knew as much about DNA as Crick did, a man who has written a book on the philosophy of science, would not feel compelled to say something on such a critical occasion. (Lear 1978, p. 38)

Although Lear’s fact-checking let him down – Crick was never knighted, and his 1966 book *Of Molecules and Men* (Crick 1966) is not on the philosophy of science – the curiousness of the incident is real, and far from unique. Over subsequent decades, Crick maintained his refusal to say anything in public about genetic engineering and never explained his silence.

Interpreting the absence of a response in a dog might be straightforward, if you are Sherlock Holmes (or rather, Arthur Conan Doyle), but drawing conclusions from the silence of a major historical figure is harder. Nevertheless, exploring Crick’s silence and the multiple reasons behind it draws attention to the absence of other key figures from the later debates that took place at Asilomar. This paper discusses two of them, Niels Jerne of EMBO, who was supposed to be a member of the organizing committee but did not attend, and Matthew Meselson of Harvard University, who was not present, despite being apparently well-qualified. These absences, their reasons and their implications suggest new lines of research into how the legacy of Asilomar was partly framed by the people who were *not* present.

## The Reasons Behind Crick’s Silence

Berg’s bemusement at Crick’s silence in Sicily flowed from the fact that he assumed that Crick would have something to say, both as a pioneer molecular biologist and as a leading scientist. That assumption, by a key figure in the debates on recombinant DNA, demands a detailed exploration, one which neither Berg nor Crick ever provided. Several different reasons – not mutually exclusive – might explain that silence; most tell us more about Crick’s interests and attitudes than they do about recombinant DNA. But Berg’s quizzical response to Crick’s apparent lack of interest highlights the fact that while contemporary historians of science and of policy might assume that debates about recombinant DNA preoccupied the scientific community in the mid-1970s, most scientists – even most molecular biologists – were actually not involved, some possibly because they were simply not interested. What is now seen as a decisive moment worthy of memorialization – and special issues of academic journals – was very much a minority affair at the time.

The simplest explanation for Crick’s silence is that his mind was elsewhere. Following the completion of the genetic code in 1967, Crick turned to other topics and by 1971 he was interested in chromosomes (Olby 2009). In May 1971, he attended a small meeting on eukaryotic DNA, where many of the latest findings on chromosome structure and organisation were discussed (Anon. 1971).^3^ During the Erice summer school a few months later, Gordon Tomkins described a potential link between RNA gene regulation mechanisms in *E. coli* and those in mammalian cells (Hershko et al. 1971). This apparently sent Crick’s mind spinning, as he put together the ideas he heard in May, Tomkins’ speculations, and recent work on histones, the proteins involved in chromosomes. Crick rapidly developed a theory of gene regulation and chromosomal structure in eukaryotes, which was rushed into print in *Nature* at the beginning of November (Crick 1971).^4^ Despite Crick’s belief that he had made a major breakthrough,^5^ his theory soon proved to be hopelessly misguided and his over-active thought processes led to a nervous collapse two weeks after the article appeared (Bretscher and Mitchison 2017). He did not fully recover for over a year.

In other words, Crick may simply have not been paying attention to the debate in Erice, given that his mind was fizzing with the implications of what he had heard from Tomkins. When Berg asked him what he thought, he may literally have had nothing to say.

It is equally possible that Crick did have views but declined to express them. Throughout his life, he was reluctant to discuss political or social matters and claimed never to have voted (Ridley 2006). He generally refused to sign petitions for worthy causes, although he did make occasional exceptions – he advocated for the release of an Israeli physiologist who was imprisoned in Syria (Edholm 1969), and for the right of molecular biologist Alexander Goldfarb, and his father, to leave the USSR – Goldfarb’s plea to the Asilomar meeting for support was simply ignored (Rogers 1977).^6^

However, during the 1960s and early 1970s, Crick did occasionally dip his toe into the complex waters of the social implications of science, before finally ceasing all public declarations at the beginning of 1972. Examination of these earlier tentative explorations by Crick shed light on his silence with regards to recombinant DNA. His approach was not particularly insightful, and when he met even the slightest criticism, he withdrew from debate. This timidity is in striking contrast with his appetite for scientific argument, which formed a key part of his way of developing his ideas (Cobb 2018).

An early example of Crick’s approach to social matters can be seen in his contribution to a small Ciba Foundation conference held in 1962 on “The Biological Future of Man” (published as *Man and His Future* – Wolstenholme 1963).^7^ The meeting involved some of the key British scientific intellectuals of the period – Peter Medawar, Jacob Bronowski, Sir Julian Huxley and J. B. S. Haldane, together with the US geneticists Joshua Lederberg and Hermann J. Muller,^8^ and focused on the apparent threats of over-population and the decline in the genetic quality of the human race, tropes that had obsessed Edwardian eugenicists (Kevles 1985). Crick’s contribution was in keeping with the overall tone of the discussion, as he explained that he wanted to “encourage by financial means those people who are more socially desirable to have more children […] The obvious way to do this is to tax children” (Wolstenholme 1963, pp. 275–276). Crick said later in the discussion that this was said “playfully” (Wolstenholme 1963, p. 284), but there is no sign that Crick was being satirical, even when he suggested it would be possible to make everyone sterile by putting a drug in their food, and then give the antidote only to people who were licenced to have children (Wolstenholme 1963, p. 275). A more accurate description of these ideas was given two decades later by Germaine Greer: “schoolboy silliness” (Greer 1984, p. 289).

Although the eugenics that was advocated by virtually all the delegates at the meeting was not racial, the policies they proposed would inevitably reinforce inequalities in society that were supposedly based on genes (Weindling 2012). This was pointed out by the French endocrinologist Marc Klein, who had been deported to Auschwitz and had witnessed the horrificsterilization “experiments” that were carried out there (Wolstenholme 1963, p. 101). Neither Crick nor anybody else responded to Klein’s point. The ideas that were discussed at the Ciba meeting were tragically typical of thinking at the time about how to deal with genetic diseases (“negative eugenics”), although Crick, like Muller and Huxley, also advocated encouraging certain groups to reproduce (“positive eugenics”) (Kay 1992; Paul 2013). By the 1980s, all these ideas had become unacceptable in most countries (Paul 2017), but at the time they were widespread, in particular amongst scientists (Donohue 2023).

The reception of the published proceedings of the Ciba meeting was overwhelmingly positive – the *Journal of the American Medical Association* claimed the book “brims with sage comments and judgments” (Reece 1966), the reviewer in *Perspectives in Biology and Medicine* praised the “lively discussion” offered by Crick and others, which contributed to the “stimulating and provocative” ideas on offer (Berrill 1964, p. 368), and *Science* found the discussions to be “often profound and original” (Crow 1965, p. 1580).

There was some contemporary criticism – in a brief review in the *Quarterly Review of Biology*, the physician Louis Lasagna skewered “the perfect nonsense” uttered by “the Nobelists and near-Nobelists” at the meeting. He felt that their comments were “sufficiently appalling” to make him appreciate that “the world is run by politicians rather than scientists” and, in a comment that fitted both Lederberg and Crick, to recommend that in future that no Nobel Prize winner “be allowed to pontificate in public about man’s problems for a five-year period after the award” (Lasagna 1965). More seriously, in 1964 the German physicist Friedrich Wagner pointed out that “[s]uch genetic visions of the ‘second cycle’ of eugenic theory remind us, however, of the ‘first cycle’ which led, a generation later, to Hitler's mass exterminations.” (Wagner 1965, p. 165). This critique formed part of growing hostility in Germany to the possibility of genetic modification (Petermann 2009).

A few years later, Crick encountered opposition, in private, to his whole approach to social questions. In October 1968, he gave a lecture at University College London (UCL) on “The Social Impact of Biology,” which was subsequently broadcast on BBC radio and was Crick’s only extended public engagement with social questions.^9^ It included a number of issues Crick felt strongly about, such as the legalization of cannabis (the year before, along with all four members of The Beatles and other celebrities, he had signed a petition in favour of a change in the law),^10^ but was focused on the widespread contemporary fears of overpopulation and on the unintended consequences of people being kept alive by new therapies. Among the apparently logical ideas Crick presented to the audience as prompts for reflection were the proposal that babies might be “legally born” only after appropriate health checks had been made in the first two days of life, while “legal death” might come into operation at eighty-five, after which euthanasia or assisted dying would be permitted.

After the lecture, Lord Annan, the Provost of UCL, Crick’s friend and the man who had invited him to speak, sent Crick a letter criticizing his naive suggestion that by teaching children about science, together with the presentation of unadorned facts, “a *new ethical system* based on modern science” would emerge, able to resolve the difficulties that were to come.^11^ In reply, Crick admitted that he was in “almost a total state of confusion about how changes occur in society.”^12^ This brief exchange was one of the rare occasions in which Crick’s approach to social and political matters was analyzed and challenged. Faced with Annan’s mild criticism, Crick apparently recognized that his understanding was inadequate – when the BBC requested permission to publish a transcript of the talk, Crick’s secretary responded that he had “reluctantly decided that he needs more time to think about a number of the topics before he commits himself to print.”^13^

Chastened by Annan’s critique, Crick concluded that he would not talk or write about social problems for a year or so.^14^ Nevertheless, he soon found himself applying his intuitive, undeveloped form of scientism to the knotty political problem of whether to attend scientific meetings in problematic places. There had been several calls to boycott conferences in countries such as Spain, because of the fascist regime, or cities, such as Chicago, because of police brutality around the 1968 Democratic Party convention. Crick had been on the sharp end of one such campaign, as the co-organizer of a NATO-sponsored Summer School that was held on the Greek island of Spetsai. After the Greek military coup of 1967, some lecturers (for example, Jim Watson and co-organizer Marianne Grunberg-Manago) refused to attend, a position that Crick considered to be “illogical” – he continued to holiday in Greece and to speak there.^15^ Faced with the threat of a further boycott of the school in 1969, Crick was able to successfully negotiate his way through the differing viewpoints and ensure that the school went ahead in 1969 (Bretscher and Mitchison 2017).^16^

Buoyed by his success, Crick decided he would draw up general principles for attending conferences in problematic places. Together with some Nobel pals – John Kendrew, Max Perutz, Fred Sanger, François Jacob, Jacques Monod and André Lwoff – Crick sent a letter to *Nature* summarizing their views (Crick et al. 1969). However, his guidelines for resolving a knotty nexus of political issues were vague and frankly useless. What seemed to Crick to be a problem that could be simply solved by principles and logic turned out to be remarkably thorny, with political subtleties that he did not perceive and was not intellectually equipped for.

This naivety was also revealed when Crick found himself peripherally involved in the growing controversy over race and IQ. In March 1969 he was contacted by William Shockley, following a brief press report of a throwaway comment he had made at the end of the UCL lecture: “[I]t is by no means clear, let me say, that all races are equally gifted.”^17^ Shockley, who was convinced that Black people were genetically inferior to whites, seized upon Crick’s comment as a sign of agreement and began sending Crick letters, putting him into contact with educationalist Arthur Jensen, whose apparently more scientific, but equally prejudiced, ideas about race and IQ interested Crick.^18^ When Crick’s friend, John Edsall, pointed out the weaknesses in Jensen’s methods, Crick illogically claimed he accepted most of these points but said he retained his confidence in Jensen’s conclusions.^19^ These private arguments, like the dispute with Annan, left Crick ill at ease, leading him to again resolve not to become involved. In October 1971, *Nature* editor John Maddox invited Crick to speak in a public debate on eugenics; Crick declined, revealing an unusual degree of self-perception:

As you know, I’ve tried to take an interest in problems concerning science and society but I’ve reluctantly come to the conclusion that I have little talent for them and no taste at all.

I have a horror of political matters, mainly because my nature leads me spontaneously towards actions which would be politically inept.^20^

However, at the beginning of 1972 Crick was one of fifty signatories, alongside Monod and Kendrew, of a petition launched by educational psychologist Ellis Page, which although it did not name Jensen or Shockley, was aimed at supporting their right to investigate race and IQ, and to receive funding (Page 1972; Serpico 2021). That signature seems to have been Crick’s last public intervention on the social implications of science.

Although Paul Berg reportedly found Crick’s silence in Erice to be exceedingly odd, it can almost certainly be explained by a combination of his preoccupation with other matters and his growing recognition of his lack of talent for discussing political and ethical issues. As Berg put it later, “[i]t just seemed like an issue that he didn’t want to have to worry or think about” (Crotty 2001, p. 94).

## Crick and Recombinant DNA

Unsurprisingly, Crick was not present at Asilomar. But he was well aware of the growing debate around recombinant DNA. He read the Berg letter (Berg et al. 1974) calling for a pause on some types of recombinant DNA experiments; when Berg spoke on the issue at the Royal Institution in London in September 1974, Crick noted it in his diary,^21^ and the eventual involvement of his close colleague, friend and office-mate Sydney Brenner in the Asilomar organizing committee would inevitably have been a topic of conversation between the two. Crick also had views on the topic. When his friend Tom Jukes dismissed concerns over manipulating *E. coli*, Crick took a different position:

I don’t entirely agree with you about the lack of dangers of putting genes into *E. coli*. I personally would be reluctant to put in SV40 genes but I would be prepared to take the risk of putting in *Drosophila* genes although even here one would have to be careful to avoid genes for drug resistance.^22^

And yet Crick said nothing about recombinant DNA in public, and if Brenner verbally broached the possibility of him attending Asilomar, Crick evidently declined. Instead, he spent early 1975 writing about the Beat poet Michael McClure (Crick 1975) and working with Peter Lawrence on an article on insect development (Crick and Lawrence 1975).

Throughout the second half of the 1970s, Crick refused invitations to write about recombinant DNA, saying variously, “I do not wish to write an article of any sort about genetic engineering,” “this is not a subject on which I have anything special to say” or, more precisely, “[t]hough the topic is not of negligible importance, I have myself played no part in the relevant discussions and have no wish to become involved in them.”^23^ Once much of the recombinant DNA dust had settled, he stated in passing in a 1983 lecture that it was highly unlikely that a laboratory leak might lead to a new plague, while fears of cloning humans were unjustified because, at the time, the technology could not even be applied to mice. However, these were technical objections; the only point at which he tangentially addressed the ethical issues was when he said it would be unwise to create 10,000 Einsteins because the human population needed variety.^24^

A decade later, Crick was slightly more forthcoming, in private at least. In 1994, a young correspondent wanted Crick’s opinion about de-extinction à la *Jurassic Park* and the possibility of controlling genetically determined traits in humans.^25^ Crick replied that he considered de-extinction to be “extremely remote and not worth considering” and produced a nuanced response on the question of human genetic engineering:

We can already alter the DNA of a very few people (with their consent) who have life-threatening diseases, and more of this is likely in the future. We have, at the moment, no good techniques for altering the DNA of the next generation (before they are born) and, even if we had, no one is keen to do this, if only because of the ethical and social problems involved.^26^

There was no hint as to what those problems might be.

## Another Notable Absentee: Niels Jerne

Crick’s absence from the arguments around recombinant DNA and the meeting at Asilomar mainly sheds light on his personality and range of intellectual interests. There is no reason to think that, had he been at Asilomar, he would have made a particularly useful contribution. At first glance, there is apparently little that can be extrapolated from his particular case to the legacies of Asilomar. However, his reluctance to be involved highlights the fact that while historians may consider that a particular debate defines a given period – in this case recombinant DNA – some actors may have either found that the issue was of peripheral significance or, as in the case of Crick, considered that it was neither valuable nor to their taste to make their views public.

A greater air of mystery surrounds another notable absentee from Asilomar – Niels Jerne, the Danish director of the Basel Institute for Immunology. In July 1974, John Kendrew, the Executive Secretary of EMBO, wrote to Berg on behalf of Jerne, who was the Chairman of the EMBO Council, saying they shared Berg’s concerns about recombinant DNA and supported the call for an international conference, expressing the willingness of EMBO to be “associated with you in this enterprise” and even “to collaborate with you organizing such a meeting” (Watson and Tooze 1981, p. 19).

A few weeks later, in August 1974, Jerne explained to *Medical Tribune* magazine that although there were problems associated with recombinant DNA, he was bullishly confident that if his laboratory were to pursue its project of introducing human genes associated with antibody production into a bacterium “this would offer advantages and be entirely harmless. There are no safety problems involved here” (Watson and Tooze 1981, p. 21). Jerne also stated that “simply calling a halt to the research does not necessarily solve any problems at this point” and that instead “[w]e must get some knowledgeable people together to determine whether we should limit this technique or not” (Watson and Tooze 1981, p. 21). Accordingly, on September 17, 1974, Berg simultaneously invited Jerne and Sydney Brenner to be part of the Asilomar organizing committee, with the aim of giving the affair a more international dimension.^27^ At the same time, EMBO encouraged its members to attend – and over twenty eventually did so (Cassata and de Chadarevian 2025).

Jerne seemed the perfect person to be involved. A senior European scientist (but, as Berg pointed out to him, neither French nor German, either of which might have excited reciprocal rivalry),^28^ Jerne had been around in the early days of molecular biology in the 1950s. He was scientifically knowledgeable and, through his role in EMBO, was used to wheeling and dealing in meetings; he also had an endless appetite for conversation (Söderqvist 2003). However, Jerne was initially reluctant to accept Berg’s invitation, saying that he had “a great number of other commitments” and that he did not “feel sufficiently competent in this particular area of molecular biology to discuss the matters you are considering with authority.”^29^ Berg pressed Jerne to change his mind: “First hand knowledge of the ‘technology’ is not a requisite; wisdom, good judgment and an appreciation of the issues are what I believe we need to help arrive at a sensible outcome.”^30^ Jerne replied that Berg’s letter had convinced him that he might “be able to play a useful rôle in the difficult task of evaluating the issues under discussion and of drafting recommendations or, at least, in coordinating these efforts with deliberations among molecular biologists in Europe, particularly through EMBO and its laboratory projects.”^31^

However, beyond encouraging members of EMBO to attend, Jerne was not involved in the activity of the organizing committee and did not attend the meeting. According to the secondary sources, the reasons behind this non-involvement are unclear and even contradictory. Either Jerne was “unable to accept” Berg’s invitation (Lear 1978, p. 113); or he “played no active role on the [organizing] committee and eventually resigned from it” (Wright 1994, p. 145); or he “did not participate in the pre-Asilomar discussions and failed to attend the conference. Consequently, he was dropped from the Organizing Committee when the official report was submitted to the NIH” (Krimsky 1982, p. 105).

On the surface, the contemporaneous explanation appears prosaic: the report to the NIH stated, as Krimsky implied, that “[t]hough Niels Jerne was appointed a member of the Organizing Committee, he did not participate in the pre-Conference activities and was unable to attend the Conference consequently he did not share in writing this report.”^32^ Around the time of Asilomar, Jerne sent Berg a “rather laconic cable” saying he could not participate in the meeting.^33^ A little over two weeks after the end of the meeting, Jerne explained his absence to Berg: “I was lying in bed with fever (probably influenza) and bronchitis and did not at that time wish this to be known.”^34^ Nevertheless, Jerne was not involved in the work of the organizing committee in January and February, before he was struck down by his malady (this was unlike Brenner, who was busy suggesting potential delegates and was involved in the Working Group on eukaryotic DNA),^35^ and it seems odd both that Jerne wanted to keep his illness private and that he waited so long before informing Berg of his situation. Nevertheless, less than three months after Asilomar, Brenner repeated this account saying that Jerne “unfortunately was ill and didn’t come.”^36^

Illness may be only part of the explanation – Jerne’s initial reluctance to accept Berg’s invitation was in marked contrast to Brenner’s enthusiastic acceptance and immediate involvement.^37^ Furthermore, a little over a year after Asilomar, in March 1976, John Tooze, the Executive Secretary of EMBO, described Jerne’s non-involvement as “very curious.” Although Tooze’s recollections of the exact circumstances were vague, there was no mention of any illness, diplomatic or otherwise:

I remember at the time (Niels) phoned and he said he wasn’t going and he told me why and it was sort of a complicated reason – he might have to be in Japan (at a) meeting, and there was this, and anyway he thought the whole damn thing was wrong, you know, and so on and so forth. It’s that sort of reason.^38^

Tooze suggested that Jerne’s view that “the whole damn thing was wrong” was due to his “philosophical attitude” which was that “a scientist ought to be allowed to do the science he wants to do, that intellectual curiosity should not be trammelled by regulations and guidelines, etc.”^39^ This agrees with Jerne’s biographer’s account of his personality, which was marked by a deep hostility to being instructed, a complete lack of desire to do anything for humanity, and a view that science (like art) was “aristocratic par excellence” (Söderqvist 2003, p. 71) – and there is no mention of Asilomar in this biography. With such firm views, it is not clear that any contribution Jerne might have made to Asilomar would have moved the debate forward or enabled any kind of agreement.

Whatever the reason behind Jerne’s absence, Berg regretted that he was not able to attend, telling him: “I am confident that had you been there your wisdom would have helped see us through some of the ‘rougher’ spots.”^40^ Brenner also considered that Jerne’s absence was a loss to the meeting, as Jerne “would have been very good on this.”^41^ Like Berg, Brenner clearly had very positive views of Jerne – he had been planning a month’s visit to Jerne’s lab in January-February 1975, but Brenner cancelled the trip because of his involvement in the work of the Asilomar organizing committee.^42^ However, given that in the same interview, Brenner expressed his despair at the belief of many US scientists that they should be allowed to do whatever they wanted (“I consider this to be right-wing talk, even if you believe in it, I don’t think you should talk that way”), it is not clear if he properly grasped Jerne’s position. Perhaps Jerne’s illness was at least partly diplomatic, enabling him to avoid participating in a process which he now felt was mistaken, and to which he would be unable to make a positive contribution; a year later, Jerne apparently considered that he did not have anything useful to say about Asilomar.^43^

## Another Non-Attender: Matthew Meselson

The selection process of delegates to Asilomar, which remains unstudied and appears to have been largely the personal choice of Paul Berg (Fredrickson 1991, p. 274), does not seem to have included pioneer molecular biologist Matthew Meselson. Not only was Meselson not amongst the attendees, there is no mention of Asilomar in the archive of his papers held at Cold Spring Harbor Laboratory, nor does there appear to be any mention in the Berg archive at Stanford of his possible attendance. This seems surprising given he not only had the requisite scientific knowledge, but also possessed an unusual degree of political acumen that might have helped advance the discussion.^44^

Meselson had expert knowledge in bacterial genetics, and a few years earlier had isolated the first restriction enzyme (Meselson and Yuan 1968); he was also an expert on biological warfare. He had campaigned against biological weapons and the use of herbicides in the Vietnam war, and his friendship with Henry Kissinger played a significant role in President Nixon’s surprise decision in November 1969 to unilaterally abandon the US biological warfare program, and to destroy bioweapon stockpiles, which led to the creation of the Biological Weapons Convention (BWC) (Meselson 2001; Perutz 2000).

In 1968, Meselson had been consulted about biological weapons by Crick, as part of his preparation for the UCL lecture. Meselson did not raise the possibility of using genetic engineering to create new weapons but described existing biological weapons – bacteria, fungi, viruses and toxins – and their effects. His conclusion was clear: “it seems to me that it would be lunacy to use or even to threaten to use a biological weapon.”^45^ In response to Meselson, Crick outlined what he described as some rather naïve tentative thoughts, arguing it would be “sensible” for countries like Sweden and Britain “to do a certain amount of research of a more or less defensive nature.”^46^ He did not there explain what he meant by “more or less,” nor outline how such research could be purely defensive. Crick’s conclusion showed that he sensed the threat that could be around the corner, although he had no remedy for it:

One final point, although I agree that at the present time biological weapons look [sic] very poor weapons, I am not convinced that this situation will necessarily always remain the same. I think what really worries me is the frightening possibility that somebody might come up with a good biological weapon. It is for this reason that I should be extremely reluctant to have, say, you or Sydney work on the development of new biological weapons!^47^

Meselson’s knowledge would have been useful for dealing with one of the key issues raised by recombinant DNA that was *not* discussed at Asilomar – the question of biological weapons. In his opening comments, on behalf of the organizing committee, David Baltimore explicitly ruled out any discussion of the matter, which he described as:

an issue which I think is very serious and which many of us have cared about for a long time, which is the potentiality to utilize this technology for biological warfare. And again, although I think it is obvious that this technology is possibly the most potent technology for biological warfare, this meeting is not designed to deal with that question. (Wright 1994, p. 149)

This position was apparently prompted by the document produced on the eve of the meeting by the Plasmid Working Group, which included the microbiologist and anti-bioweapons campaigner Richard Novick. The document concluded with a clear statement on the threat of bioweapons, which the group wanted to include in the final declaration of the conference:

We believe that perhaps the greatest potential for biohazards involving alteration of microorganisms relates to possible military applications. We believe strongly that construction of genetically altered microorganisms for any military purpose should be expressly prohibited by international treaty, and we urge that such prohibition be agreed upon as expeditiously as possible.^48^

According to historian of science Susan Wright, the tapes of the hectic final session of the conference reveal “some participants repeatedly trying to insert a statement to the effect that certain experiments were deemed so dangerous that they should not be performed under any circumstances” and meeting the firm opposition of Berg and Baltimore (Wright 1994, p. 156). When asked why the statement on bioweapons from the Plasmid Working Group had not been included in the statement from the organizing committee that was being discussed and voted on, Baltimore explained their thinking:

That was a conscious decision, based on the demonstrable split in opinion at this meeting about whether this is a philosophical question or a practical question. There were a number of people who told us at significant length that […] this was a philosophical question of rights of free inquiry. (Wright 1994, p. 156)

An apparent compromise was found by Berg, who rephrased the Plasmid Working Group statement as “there is a class of experiments that should not be done at all irrespective of the type of containment available today.” This statement, which left it open whether in the future such experiments would be permissible, was adopted with only a few dissenters (Wright 1994, p. 156).

The issue of bioweapons did not go away. Shortly after Asilomar, Baltimore reported to the rest of the organizing committee that he had said in passing at a meeting that “a good idea would be an international treaty” to control biological warfare.^49^ Afterwards, he explained, he was called by “a guy” from the US Arms Control and Disarmament Agency (ACDA) who informed him that the 1972 BWC also covered “any development of new biological weaponry.”^50^ (The BWC was eventually signed by eighty-five nations, including the USA and the USSR, and came into effect a month after the end of Asilomar; Wheelis et al. 2002). Baltimore also said that in a subsequent discussion with Robert Mikulak of the ACDA, it was suggested that it might be possible to get a specific interpretation of the BWC “that would say it outlaws use of recombinant DNA technology for purposes of developing biological weaponry.”^51^ This position was supported the following year at the U.N. Conference of the Committee on Disarmament, and was adopted in 1980 by the First Review Conference of the parties to the BWC, held in Geneva. However, US military interest in recombinant DNA was growing (Wright and Sinsheimer 1983), and it is now known that, even before Asilomar, the USSR had launched a program of bioweapons development using recombinant DNA, known as FERMENT (Leitenberg and Zilinskas 2012).

## Conclusion

The absences of Crick, Jerne and Meselson from Asilomar each had different consequences, each of which can be outlined by brief counterfactuals.

In Crick’s case, his absence is easy to understand, and his presence would probably have had little impact. Not only did he feel unconfident about venturing into issues with political implications, he had no practical knowledge of the techniques being deployed, nor did he have any specialist understanding of microbiology or epidemiology. Along with his self-reported horror of political matters, his silence in Erice and afterwards suggests that he recognized he was simply not qualified to comment.^52^

Jerne, who apparently missed the meeting through illness, was well-qualified to be involved in the process, but his views about the position of science and the independence of scientific inquiry might have reinforced the views within the meeting, expressed in particular by Jim Watson and Joshua Lederberg, opposing both the pause introduced by the Berg letter and the idea of any kind of declaration being adopted by the meeting (Wright 1994). Given that the renown of Asilomar is largely based on the meeting’s apparent ability to overcome differences, Jerne’s presence might have undermined this.

Meselson’s absence draws attention to one of the most significant but under-studied aspects of the meeting – the threat posed by the potential application of recombinant DNA to biological weapons developments. This issue, which was deliberately taken off the agenda by the organizers and sidestepped in the final declaration, was one of the most significant implications of recombinant DNA that was not discussed at Asilomar. Despite the assurances of the BWC, the USSR at least was able to apply recombinant DNA to bioweapons development successfully; other countries may well have done the same (Wheelis et al. 2002).

These examples highlight the fact that while perceptions of Asilomar might suggest that debates about recombinant DNA preoccupied the scientific community in the mid-1970s, most scientists – even most molecular biologists – were not involved. They may have had opinions, but they did not express them and were not actively engaged in the arguments. Some may have had opinions that were unwelcome. Still others may not have been concerned at all. Understanding scientists’ lack of involvement in Asilomar and its consequences may provide a useful angle of attack in future explorations of the legacy of the meeting and the mechanics of the relatively informal processes that apparently underpinned the selection of attendees.

One issue that could usefully be explored would be the lack of involvement of those at the other end of the academic and political seniority scale to the three examples explored here. A year after the conference, Brenner remarked that “the kind of real discussions on this issue were done by pretty old people. I mean, they weren’t young graduate students; they were all very quiet.”^53^ Brenner was particularly concerned by the absence of opponents of genetic engineering from Science for the People (SftP), who submitted a document to Asilomar (Watson and Tooze 1981, p. 49), which he claimed, “most people just read and threw away.”^54^ For complex reasons, no one from SftP was present at Asilomar; according to SftP member Jonathan King it would have been “a set-up, to have one left guy at a conference of a hundred and fifty people.”^55^ The result was that the debates, although already complex, lacked a whole viewpoint that would subsequently become significant as the public became aware of the new technique (Cobb 2022).

Asilomar is one of the most intensely studied moments in the history of 20th century biology, and its legacy is regularly invoked with each notable anniversary and each new apparently alarming technological development. And yet we are still far from having a full understanding of the way in which the debates were framed by those who were present, and those who were not.

## Data Availability

No datasets were generated or analyzed during the current study.

## Footnotes

1 Berg, P. 2000. *A Stanford professor’s career in biochemistry*,* science*,* politics*,* and the biotechnology industry.* An oral history conducted in 1997 by Sally Smith Hughes. Berkeley: Regional Oral History Office, The Bancroft Library, University of California.

2 All details of this school are taken from the file of documents in Francis Crick (1916–2004): archives, Wellcome Collection, London (hereafter: Crick-Wellcome), PP/CRI/E/1/19/8.

3 The papers associated with the meeting can be found at Crick-Wellcome PP/CRI/E/1/19/6.

4 See drafts and notes at Crick-Wellcome PP/CRI/H/5/1.

5 Crick to Maddox, 3 September 1971, Crick-Wellcome, PP/CRI/D/2/27.

6 Goldfarb to Crick, 24 January 1981, Crick-Wellcome, PP/CRI/D/1/4/7.

7 All details and quotes taken from Wolstenholme (1963).

8 Muller was unwell so did not attend; his contribution was read out.

9 Crick always spoke from notes, not a written script. All that survives in the public domain for this lecture is Crick, “Notes: The social impact of biology,” undated [1968], Crick-Wellcome, PP/CRI/E/1/16/13/1. The lecture was recorded by the BBC and broadcast on Radio 3 on 19 February 1969: *Radio Times*, 15 February 1969. The BBC holds a copy of the recording and I have been able to listen to it.

10 *The Times*, 24 July 1967.

11 Annan to Crick, 4 November 1968, Crick-Wellcome, PP/CRI/D/1/1/1.

12 Crick to Annan, 8 November 1968, The Papers of Noel Gilroy Annan, King’s College, Cambridge, NGA/5/1/203/1.

13 Miller to Crick, 24 January 1969; Crick secretary to Miller, 23 January 1969; Crick to Miller, 6 February 1969, Crick-Wellcome, PP/CRI/D/1/1/13.

14 Crick to Shockley, 2 April 1969, Crick-Wellcome, PP/CRI/D/2/39.

15 Crick to Grunberg-Manago, 30 August 1967; Grunberg-Manago to Crick, 11 September 1967; Crick to Evangelopoulos, 29 November 1967, Crick-Wellcome, PP/CRI/E/1/17/7/1; Crick to Berg, 22 December 1967, Crick-Wellcome, PP/CRI/E/1/17/7/2. Crick to Evangelopoulos, 14 March 1968, Crick-Wellcome, PP/CRI/E/1/17/7/1.

16 All documents relating to this affair can be found in Crick-Wellcome, PP/CRI/E/1/17/7/3. See also Crick to Evangelopoulos, 10 April 1969; Crick-Wellcome, PP/CRI/E/1/17/7/1; Crick to Sorokos, 24 January 1972, Crick-Wellcome, PP/CRI/D/1/2/2 and Gros to Crick, 21 May 1969, Crick-Wellcome, PP/CRI/D/1/1/7.

17 Shockley to Crick, 17 March 1969, Crick-Wellcome, Crick-Wellcome, PP/CRI/D/2/39. This quote is taken from the BBC recording of the lecture. A brief quote of this phrase appeared in *Riverside Press*, 6 March 1969, following the broadcast. A cutting of this article, together with Crick’s correspondence with Shockley, can be found at Crick-Wellcome, PP/CRI/D/2/39.

18 For example, Crick to Jensen, 21 February 1972, Crick-Wellcome, PP/CRI/D/1/2/5.

19 Edsall to Crick, 5 March 1971; Crick to Edsall, 29 March 1971, Crick-Wellcome, PP/CRI/D/2/14.

20 Maddox to Crick, 20 October 1971; Crick to Maddox, 25 October 1971, Crick-Wellcome, PP/CRI/E/2/10.

21 Crick to Jukes, 24 July 1974, Crick-Wellcome, PP/CRI/D/1/2/5. Crick, diary entry for 2 September 1974, Crick-UCSD, MSS 660, Box 14, Folder 20.

22 Jukes to Crick, 19 July 1974; Crick to Jukes, 24 July 1974, Crick-Wellcome, PP/CRI/D/1/2/5.

23 Crick to Teresi, 9 November 1976, Crick-Wellcome, PP/CRI/D/1/3/16. Crick to Ubell, 21 September 1976, Crick-Wellcome, PP/CRI/D/1/3/18. Crick to Dallas, 7 March 1977, Crick-Wellcome, PP/CRI/D/3/4. These were invitations from *Science Digest*, *The Sciences*, and *The Southern California Law Review*, respectively.

24 See file of cuttings and correspondence at Crick-Wellcome, PP/CRI/K/2/10.

25 Raab to Crick, undated [August 1994], Crick-Wellcome, PP/CRI/J/1/4/16. Crick to Raab, 2 September 1994, Crick-Wellcome, PP/CRI/J/2/14/3.

26 Crick to Raab, 2 September 1994, Crick-Wellcome, PP/CRI/J/2/14/3.

27 Berg to Jerne, 17 September 1974, Paul Berg papers, Stanford Special Collections and University Archives SC0358 (hereafter Berg-Stanford), Box 13, Correspondence 1974-75 I-R. Berg to Brenner, 17 September 1974, Berg-Stanford, Box 13, Correspondence 1974–75 A-C.

28 Berg to Jerne, 16 October 1974, MIT Oral History Program on Recombinant DNA, MC-0100 (hereafter MIT-OHP), Box 40, Folder 615.

29 Jerne to Berg, 4 October 1974, Berg-Stanford, Box 13, Correspondence 1974-75 I-R.

30 Berg to Jerne, 16 October 1974, MIT-OHP, Box 40, Folder 615.

31 Jerne to Berg, 12 November 1974, Berg-Stanford, Box 13, Correspondence 1974-75 I-R.

32 Report of the Organizing Committee of the Asilomar Conference on Recombinant DNA Molecules, CSHL-SB, SB/4/1/24.

33 Jerne to Berg, 12 March 1975, MIT-OHP, Box 40, Folder 615.

34 Jerne to Berg, 12 March 1975, MIT-OHP, Box 40, Folder 615.

35 Novick to Murray, 28 January 1975, Sydney Brenner Collection, Cold Spring Harbor Library and Archive (hereafter CSHL-SB), SB/4/1/25.

36 Interview with Brenner by Weiner, May 21, 1975, pp. 58–59, MIT-OHP, Box 14, Folder 26.

37 Brenner to Berg, 24 September 1974, Berg-Stanford, Box 13, Correspondence 1974–75 A-C.

38 Interview with Tooze by Weiner, March 26, 1976, p. 40, MIT-OHP, Box 14, Folder 162.

39 Interview with Tooze by Weiner, March 26, 1976, p. 40, MIT-OHP, Box 14, Folder 162.

40 Berg to Jerne, 25 March 1975, Berg-Stanford, Box 13, Correspondence 1974-75 I-R.

41 Interview with Brenner by Weiner, May 21, 1975, pp. 58–59, MIT-OHP, Box 14, Folder 26.

42 Brenner to Jerne, 21 November 1974, CSHL-SB, SB/1/1/302.

43 Interview with Tooze by Weiner, March 26, 1976, p. 41, MIT-OHP, Box 14, Folder 162.

44 I have endeavored to discover from Meselson why he was not present.

45 Meselson to Crick, 21 October 1968, Crick-Wellcome, PP/CRI/D/1/1/13. Crick’s letter, written while travelling, has been lost.

46 Crick to Meselson, 20 November 1968, Crick-Wellcome, PP/CRI/D/1/1/13.

47 Crick to Meselson, 20 November 1968, Crick-Wellcome, PP/CRI/D/1/1/13.

48 Royston C. Clowes, Stanley N. Cohen, Roy Curtiss III, Stanley Falkow and Richard Novick, Proposed Guidelines on Potential Biohazards Associated with Experiments Involving Genetically Altered Microorganisms, 24 February 1975, p. 19, CSHL-SB, SB/4/1/25.

49 Baltimore to Berg, Roblin, Singer and Brenner, 28 April 1975, CSHL-SB, SB/4/1/36.

50 Baltimore to Berg, Roblin, Singer and Brenner, 28 April 1975, CSHL-SB, SB/4/1/36.

51 Baltimore to Berg, Roblin, Singer and Brenner, 28 April 1975, CSHL-SB, SB/4/1/36.

52 Perhaps in recognition of Crick’s non-involvement in the discussions before, at, and after Asilomar, Watson and Tooze dedicated their collection about the recombinant DNA controversy, *The DNA Story*, to Crick (Warson and Tooze 1981).

53 Interview with Brenner by Weiner, May 21, 1975, pp. 86–87, MIT-OHP, Box 14, Folder 26.

54 Interview with Brenner by Weiner, May 21, 1975, p. 87, MIT-OHP, Box 14, Folder 26.

55 Interview with King, August 26 1975, MIT-OHP, Box 9, Folder 108.
